# Exploring the mechanisms under Zuogui Pill’s treatment of ischemic stroke through network pharmacology and *in vitro* experimental verification

**DOI:** 10.3389/fphar.2023.1153478

**Published:** 2023-06-22

**Authors:** Li Li, Yan Liu, Yawei Zheng, Jian Zhu, Dan Wu, Xiaohui Yan, Changyin Li, Minghua Wu, Wenlei Li

**Affiliations:** ^1^ Department of Neurology, Jiangsu Province Hospital of Chinese Medicine, Affiliated Hospital of Nanjing University of Chinese Medicine, Nanjing, China; ^2^ The First Clinical Medical College, Nanjing University of Chinese Medicine, Nanjing, China; ^3^ Department of Endocrinology, Nanjing First Hospital, Nanjing Medical University, Nanjing, China; ^4^ Department of Clinical Pharmacology, Jiangsu Province Hospital of Chinese Medicine, Affiliated Hospital of Nanjing University of Chinese Medicine, Nanjing, China

**Keywords:** zuogui pill, ischemic stroke, network pharmacology, neurite outgrowth, SH-SY5Y cells

## Abstract

Due to its high mortality, incidence and disability rates, ischemic stroke poses heavy economic burdens to families and society. Zuogui Pill (ZGP) is a classic Chinese medicine for tonifying the kidney, which is effective for the recovery of neurological function after ischemic stroke. However, Zuogui Pill has not been evaluated for its potential effects on ischemic strokes. Using network pharmacology, the research aimed to explore the mechanisms of Zuogui Pill on ischemic stroke, which were further validated in SH-SY5Y cells injured by oxygen and glucose deprivation/reperfusion (OGD/R). Network analysis of Zuogui Pill identified 86 active ingredients and 107 compound-related targets correlated with ischemic stroke. Additionally, 11 core active compounds were obtained, such as Quercetin, beta sitosterol, and stigmasterol. Most of the compounds have been proven to have pharmacological activities. Based on pathway enrichment studies, Zuogui Pill may exert neuroprotection through MAPK signaling, PI3K-Akt signaling and apoptosis, as well as enhance neurite outgrowth and axonal regeneration effect via mTOR signaling, p53 signaling and Wnt signaling pathways. *In vitro* experiment, the viability of ischemic neuron treated with Zuogui Pill was increased, and the ability of neurite outgrowth was significantly improved. Western blot assays shown that the pro-neurite outgrowth effect of Zuogui Pill on ischemic stroke may be relate to PTEN/mTOR signal pathway. The results of the study provided new insights into Zuogui Pill’s molecular mechanism in treatment of ischemic stroke, as well as clinical references for its use.

## Introduction

Chinese adults suffer from ischemic stroke most often, which leads to death and disability (Sun et al., 2021). It is estimated that approximately 13.7 million people suffer from stroke each year globally, and about 87% are related to ischemia (Saini et al., 2021). In acute ischemic stroke patients within the time window, intravenous thrombolysis and intravascular therapy are the most effective treatment measures (Suzuki et al., 2021). However, fewer than 5% of the patients were able to undergo effective thrombolysis and thrombectomy limited by the relatively short treatment time window (Saini et al., 2021). The majority of survivors suffer from serious neurological deficit symptoms, which severely impair their quality of life and burden society as a whole. In addition to vascular recanalization and neuroprotective strategies in the acute phase (Paul and Candelario-Jalil, 2021), neuronal regeneration and axonal repair run through the whole process of ischemic stroke, and play an even more important role during the recovery process. The prognosis of ischemic stroke can be significantly improved by promoting axonal sprouting, forming new collateral and synapse of damaged neurons, and reconstructing neural networks (Regenhardt et al., 2020; Takase and Regenhardt, 2021). However, no effective drug existed to promote nerve regeneration. The development of novel, safe and efficient medications for the regeneration and remodeling of ischemic stroke is highly required.

Traditional Chinese medicine categorizes ischemic stroke as “stroke” (Yu et al., 2016). The main pathogenesis is deficiency of yin and essence of the liver and kidney. On this basis, wind, fire, phlegm, Qi and blood and other pathological products cause cerebrovascular obstruction and brain marrow damage (Yu et al., 2016). Tonifying the kidney to generate the marrow, and filling the brain with marrow can promote the repair of the brain marrow and the recovery of neurological function (Hu et al., 2012; Luo et al., 2020). Zuogui pill (ZGP) is a traditional medicine prescription for tonifying kidneys and yins. Zhang Jingyue, a physician during the Ming Dynasty, created it to nourish the kidney, fill the essence, nourish the kidney and generate marrow (Chen et al., 2010). In the previous clinical and experimental studies, ZGP has shown definited effects on ischemic stroke (Li et al., 2019; Liu et al., 2022). The research about the mechanism of ZGP showed that it has the effects of protecting brain cells (Liu et al., 2022), promoting nerve regeneration and remodeling after ischemic stroke (Wang et al., 2011). However, there is still not a clear understanding of the material basis of ZGP and the potential molecular mechanisms that underlie its ability to promote neural regeneration and functional recovery.

The technology of network pharmacology focuses on the relationship between drug molecules, action mechanisms, and disease targets based on the construction of biological networks (Wang et al., 2021). Integrity and systematicity are key characteristics of this technology, which are in sync with traditional Chinese medicine’s holistic approach and personalized treatment based on symptoms (Jiao et al., 2021). Therefore, it is widely used to predict the correlation between the pharmacodynamic components and the action mechanism of complex Traditional Chinese medicine (Wang et al., 2021; Yang et al., 2022). The study explored ZGP’s mechanism in multi-target, multi-channel treatment of ischemic stroke using the network pharmacology method. Additionally, part of the mechanism was confirmed by *in vitro* experiments, which provided a biological basis for treating ischemic stroke with “Bushen Shengsui” Chinese medicine.

## Materials and methods

### Database construction of ZGP

**FIGURE 1:**
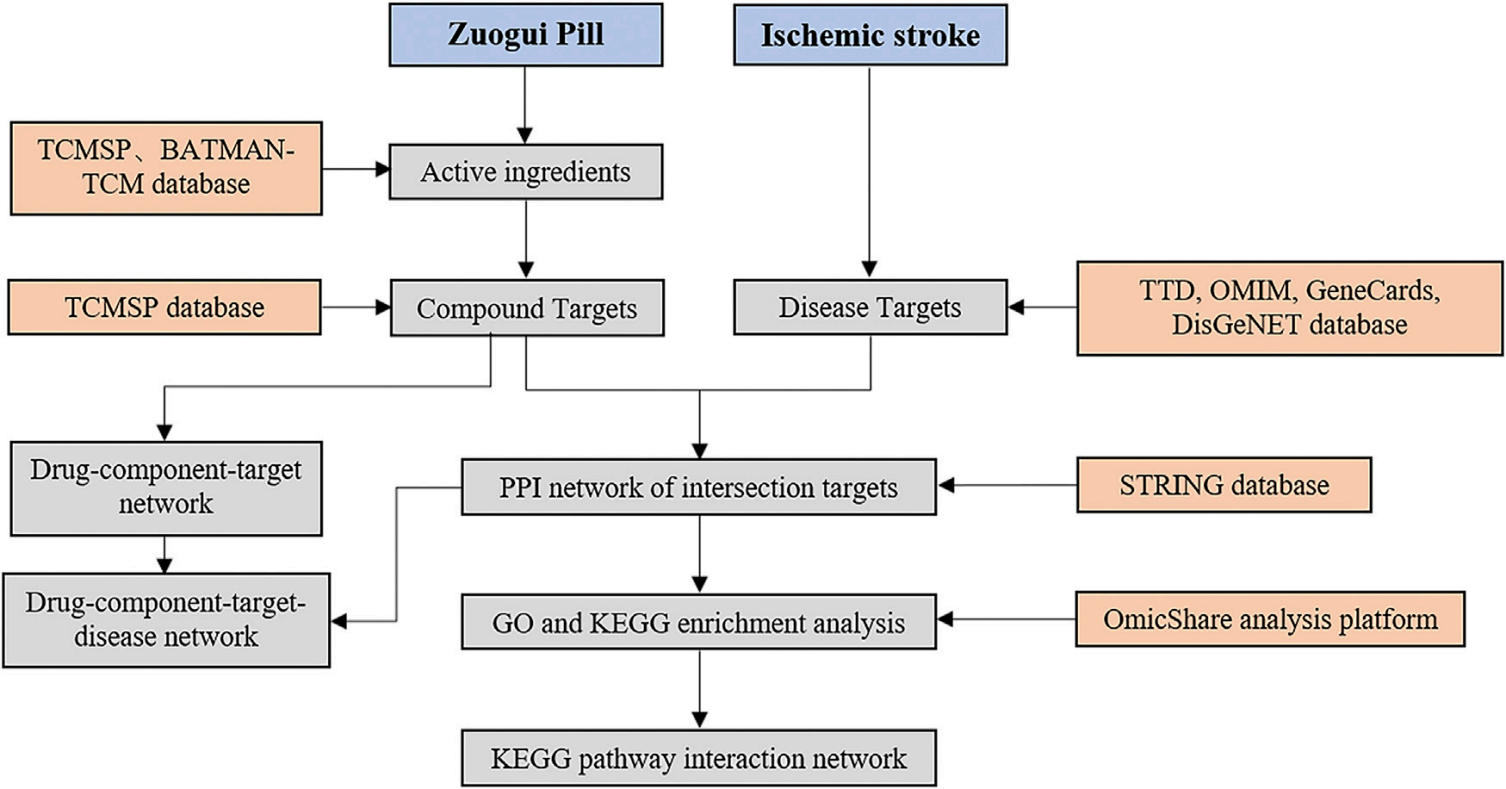
The work procedure to explain the mechanism of ZGP.

Compounds in ZGP (Rehmannia glutinosa, Yam, Cornus officinalis, Achyranthes bidentata, Medlar, Dodder, Tortoise shell glue, Antler glue) were search in TCMSP (http://lsp.nwu.edu.cn/tcmsp.php) and BATMAN-TCM (http://bionet.ncpsb.org/batman-tcm/). The rate and degree of drug absorption in the body is referred to as oral bioavailability (OB). A compound’s drug-likeness (DL) is defined as its similarity to a known drug. For the purpose of screening potential compounds, the active components with OB ≥ 30% and DL ≥ 0.18 were chosen. Figure 1 depicts the procedure for elucidating the ZGP mechanism.

### Prediction of compound targets for ZGP

Search compound targets through TCMSP database, screen and eliminate the duplicate targets. The gene names and UniProt numbers of each target were converted into target abbreviations using the UniProt database (http://www.uniprot.org/uniprot/). Get rid of targets that are repetitive, not human or standard. Cytoscape 3.8 software was used to import the ZGP’s primary active components and their targets to create a “Component-Target” network diagram. The targets and chemical components that were represented by the nodes in the network diagram. Interactions were represented by the edges. The degrees reflected the number of edges that combined a node, and function and degree values were positively associated. The information regarding degree centrality (DC), medium centrality (BC), and near centrality (CC) was obtained by evaluating the topological properties with the “CytoNCA” function.

### Targets of ZGP against ischemic stroke prediction

By utilizing the phrase “ischemic stroke,” disease targets were found in the TTD database (http://db.idrblab.net/ttd/), DisGeNET database (https://www.disgenet.org), GeneCards database (https://www.genecards.org), and OMIM database (http://omim.org/). With UniProt, duplicate targets were eliminated. The targets of the active ZGP components were compared to the targets of ischemic stroke by using the Venny platform (http://bioinfogp.cnb.csic.es/Tools/Venny/), and the targets of ZGP for treating ischemic stroke were determined.

### Construction of drug-component-target-disease network

Each drug and drug active ingredient of ZGP was defined as “drug-component network” in Excel file format. The drug active ingredient and active ingredient target were defined as “component-target network” in Excel file format, and the disease and disease potential target were defined as “disease-target network” in Excel file format. The above three files were imported into Cytoscape 3.8 software, and “drug-component-target-disease network” of ZGP was obtained by using merge function of the software. The “CytoNCA” function was used to analyze the topological properties.

### Analysis of the protein-protein interaction (PPI) network

For the purpose of PPI analysis, the common targets of ZGP and ischemic stroke were imported into the STRING database (version 11.0, https://string-db.org/). For the PPI network construction, the interacting proteins with a high confidence score of ≥0.700 were chosen. Cytoscape 3.8 was used to import the aforementioned data, create a target PPI network diagram, and screen the core network. In order to identify potential hub targets, the “CytoNCA” function was utilized to examine the topological properties.

### GO and KEGG enrichment analysis

For further analysis, the intersection targets were uploaded to the OmicShare analysis platform. OmicShare was used to analyze gene ontology (GO) data, the Kyoto Encyclopedia of Genes and Genomics (KEGG) pathway. The biological process (BP), the cellular component (CC), and the molecular function (MF) are all included in the GO enrichment analysis. By setting the *p*-value, the GO enrichment results were obtained, and the top 20 categories were chosen for visualization. The KEGG pathway interaction network was established for the enrichment results of KEGG pathways, and its topological properties were analyzed by using the function of “CytoNCA” to uncover the potential key pathways.

### Molecular docking analysis

Molecular docking aims to simulate the docking between small molecule ligands and large molecule proteins, and the docking results are evaluated by binding energy (affinity). Lower binding energy indicates better molecular docking binding effect. The 3D structure of the compounds were obtained from TCMSP database (http://tcmspw.com/tcmsp.php), and the structure of target proteins were obtained from RCSB PDB (https://www.rcsb.org/). AutoDock Tools 1.5.6 was applied to perform molecular docking and predict the binding energy of compounds and proteins. Finally, PyMOL software was used to visualize the optimal docking results.

### Chemicals and reagents for pharmacological verification

One prescription of ZGP comprises Rehmannia glutinosa 24 g, Fried yam 12 g, Lycium barbarum 12 g, Cornus officinalis 12 g, Sichuan Achyranthes 9 g, Dodder seed 12 g, Antler gum 12 g, and Tortoiseshell gum 12 g. The ratio of these herbs was 8:4:4:4:3:4:4:4. All of these ingredients were acquired from China and obtained from the pharmacy of Jiangsu Province Hospital of Chinese Medicine. The Chinese Pharmacopoeia 2015 was employed to verify the ZGP. A voucher specimen (2021–0527) was placed at the Central Laboratory of Jiangsu Province Hospital of Chinese Medicine. The detailed approaches for preparing and quality assurance of ZGP were as follows: A total of 10 prescriptions of ZGP (1050 g) were prepared, mixtures of components in ZGP were soaked with 1000 mL water for 30 min, then decocted twice for 30 min. The liquid was decocted twice, collected, concentrated to a density of about 1.3, and dried under vacuum at 60°C. Finally, the extract was crushed to obtain ZGP-dried powder (362 g). The powder was preserved in aliquots at −20°C and dissolved in a cell culture medium to create working solutions for bioassays. SH-SY5Y human neuroblastoma cells were supplied by the Chinese Academy of Sciences Stem Cell Bank. All-trans-retinoic acid (RA) was purchased from SIGMA (Unite State). Brain-derived neurotrophic factor (MCE, Unite State). The anti- GAP43, p-S6, mTOR, PTEN, and GAPDH were purchased from CST (Unite State).

### Cell culture and treatment

The Chinese Academy of Sciences Stem Cell Bank supplied the SH-SY5Y human neuroblastoma cells. In DMEM-F12, SH-SY5Y cells were treated with 10% FBS, 100 U/mL penicillin, and 100 mg/mL streptomycin and cultivated at 37°C in a 5% CO2 environment. SH-SY5Y cells were cultivated in 6 cm culture plates at a density of 1.5×10^6^ and separated into control and all-trans-retinoic acid (RA) induction cohort. The control cohort was cultured normally, while the RA induction cohort was treated with 5 μmol/L RA to induce cells differentiation into mature neurons (Jahn et al., 2017). The culture medium was changed once a day for three consecutive days. Then, oxygen-glucose deprivation/reoxygenation (OGD/R) procedure was used to treat the differentiated SH-SY5Y cells to mimic cerebral ischemia reperfusion injury *in vitro* (Zhi et al., 2020; Sun et al., 2022). The differentiated SH-SY5Y cells induced by RA were maintained in glucose-free EBSS (Early’s Balanced Salt Solution) medium and kept in a three-gas incubator (O2 1%, CO2 5%, N2 94%) for 4 h. Subsequently, the cells were relocated to a normal culture medium and preserved in a normal incubator for the following experiments. ZGP was dissolved in normal culture medium and prepared into working solution with a series of concentrations (0.14–5.12 mg/mL). The cells were divided into the following cohorts for different experimental purposes: normal control (NC), differentiated SH-SY5Y cells induced by RA (RA), oxygen-glucose deprivation/reoxygenation (OGD), OGD + ZGP. To detect the impacts of ZGP on cell viability, the cells were treated with ZGP for 24 h. To observe the impacts of ZGP on neurite outgrowth and expression of target protein, the cells were supplemented with them for 72 h.

### Cell viability assay

The viability of the cells was determined with the help of the CCK-8 test. In 96-well plates, the differentiated SH-SY5Y cells were seeded at a density of 3 × 10^4^ cells per well in 100 μL of media. The cells were distributed into four cohorts: normal control (NC), differentiated SH-SY5Y cells induced by RA (RA), oxygen-glucose deprivation/reoxygenation (OGD), OGD + ZGP. For the ZGP cohorts, the cells were supplemented with ZGP at a density from 0.14 to 5.12 mg/mL for 24 h. The cells received 10 μL of CCK-8 reagent after undergoing a variety of treatments, and the optical density (OD) was measured at 490 nm after an additional 4 h of cultivation. Cell viability was obtained by calculating the ratio of OD value of each cohort to that of control cohort (Cell viability = OD value of all cohorts/OD value of the NC cohort ×100%).

### Neurite outgrowth analysis

The SH-SY5Y cells were seeded at a density of 5 ×10^5^ cells per well in 500 μL of media in 24-well plates. After inducing differentiation by ATRA and undergoing OGD/R injury, the cells were divided into the following groups: normal control (Control), oxygen-glucose deprivation/reoxygenation (OGD), OGD + ZGP (0.16, 0.32, 0.64 mg/mL). A treatment with 50 ng/mL of brain-derived neurotrophic factor (BDNF) was used as positive controls for neurite outgrowth (Dedoni et al., 2012; Agapouda et al., 2022). After being treated with different methods for 72h, the growth status of cell neurites was analyzed by measuring the length of neurites. The images of cells and neurites were obtained using a ZEISS LSM-710 Confocal Microscope (ZEISS Microsystems, Germany). Six high-power fields (40×) were detected from each well of different cohorts. The length of 10 neurites was measured per visual field using the ImageJ software, and the quantitative analysis was performed using Prism 9.0 software.

### Western blot analysis

For Western blotting, the protein was extracted from cells to detect the target protein expression. After being treated with different methods, the cells were retrieved and extracted protein with RIPA lysate. The BCA method was used to determine the protein concentration. Conditions for SDS-PAGE gel electrophoresis: 80 V for 30 min and 120 V for 60 min. Conditions for membrane conversion: 250 mA of constant current for 90 min, completed on ice, and sealed for 1 hour at room temperature (25°C) with skimmed milk powder. A primary rabbit anti-GAP43 (1:1000), rabbit anti-p-S6 (1:1000), mouse anti-mTOR (1:5000), GAPDH (1:20,000), and rabbit anti-PTEN (1:2000) were used to preserve the membranes overnight at 4°C. After being treated with secondary goat anti-rabbit IgG (1:5000) or goat anti-mouse IgG (1:2000) antibodies for 1 hour and electrochemiluminescence (ECL) reagents for 30 s to 2 minutes, the membranes were exposed to Kodak film (Japan). The IOD proportion was measured and evaluated employing the ImageJ program to display the data.

### Statistical analysis

For statistical analysis, the software SPSS26.0 and Graphpad Prism 8 were utilized. All data were tested for normality using the Shapiro-Wilk test. Normally distributed parameters were expressed as mean±S.D. The one-way ANOVA and the Tukey multiple comparisons test were used to identify group differences. LSD-t test was used for pairwise comparison. The statistical significance was described as *p < 0.05, **p < 0.01, ***p < 0.001, ****p < 0.0001.

## Results

### ZGP active components screening

Eighty-six active components of ZGP were retrieved from the TCMSP and TCMID databases in accordance with the two screening criteria of OB value (≥30%) and DL index (≥0.18). These components include 2 components in SDH (Rehmannia glutinosa), 16 components in SY(Fried yam), 20 components in SZY(Cornus officinalis), 4 components in CNX (Sichuan Achyranthes), 48 components in GQ (Lycium barbarum), and 12 components in TSZ (Dodder seed). The six main components in GBJ (Tortoiseshell gum) and the two main components in CNX (Sichuan Achyranthes) were removed because they did not meet the screening requirements. The most important ZGP active components were shown in Table 1.

**TABLE 1:** The active components of ZGP.

Mol ID	Molecule name	OB (%)	DL	Source
MOL000098	quercetin	46.4	0.275	CHUANNIUXI, GOUQIZI, TUSIZI
MOL000184	NSC63551	39.3	0.759	TUSIZI
MOL000310	Denudatin B	61.5	0.378	SHANYAO
MOL000322	Kadsurenone	54.7	0.378	SHANYAO
MOL000354	isorhamnetin	49.6	0.306	TUSIZI
MOL000358	beta-sitosterol	36.9	0.751	SHANZHUYU, CHUANNIUXI, GOUQIZI, TUSIZI
MOL000359	sitosterol	36.9	0.751	SHUDIHUANG, SHANZHUYU
MOL000422	kaempferol	41.9	0.241	TUSIZI
MOL000449	Stigmasterol	43.8	0.757	SHUDIHUANG, SHANZHUYU, SHANYAO, GOUQIZI
MOL000546	diosgenin	80.9	0.810	SHANYAO
MOL000554	gallic acid-3-O-(6′-O-galloyl)-glucoside	30.3	0.675	SHANZHUYU
MOL000953	CLR	37.9	0.677	SHANYAO, GOUQIZI, TUSIZI
MOL001323	Sitosterol alpha1	43.3	0.784	GOUQIZI
MOL001494	Mandenol	42.0	0.193	SHANZHUYU, GOUQIZI
MOL001495	Ethyl linolenate	46.1	0.197	SHANZHUYU, GOUQIZI
MOL001558	sesamin	56.5	0.827	TUSIZI
MOL001559	piperlonguminine	30.7	0.180	SHANYAO
MOL001736	(−)-taxifolin	60.5	0.273	SHANYAO
MOL001771	poriferast-5-en-3beta-ol	36.9	0.750	SHANZHUYU
MOL001979	LAN	42.1	0.748	GOUQIZI
MOL002320	Gamma-Sitosterol	36.9	0.750	GOUQIZI
MOL002773	Beta-Carotene	37.2	0.580	GOUQIZI
MOL002879	Diop	43.6	0.392	SHANZHUYU
MOL002883	Ethyl oleate (NF)	32.4	0.191	SHANZHUYU
MOL003137	Leucanthoside	32.1	0.781	SHANZHUYU
MOL003578	Cycloartenol	38.7	0.781	GOUQIZI
MOL005043	campest-5-en-3beta-ol	37.6	0.715	TUSIZI
MOL005360	malkangunin	57.7	0.626	SHANZHUYU
MOL005406	atropine	46.0	0.193	GOUQIZI
MOL005429	hancinol	64.0	0.373	SHANYAO
MOL005430	hancinone C	59.0	0.390	SHANYAO
MOL005435	24-Methylcholest-5-enyl-3belta-O-glucopyranoside_qt	37.6	0.717	SHANYAO
MOL005438	campesterol	37.6	0.715	SHANYAO, GOUQIZI, TUSIZI
MOL005440	Isofucosterol	43.8	0.758	SHANYAO, TUSIZI
MOL005458	Dioscoreside C_qt	36.4	0.871	SHANYAO
MOL005461	Doradexanthin	38.2	0.537	SHANYAO
MOL005463	Methylcimicifugoside_qt	31.7	0.237	SHANYAO
MOL005465	AIDS180907	45.3	0.773	SHANYAO
MOL005481	2,6,10,14,18-pentamethylicosa-2,6,10,14,18-pentaene	33.4	0.240	SHANZHUYU
MOL005486	3,4-Dehydrolycopen-16-aL	46.6	0.491	SHANZHUYU
MOL005489	3,6-Digalloylglucose	31.4	0.663	SHANZHUYU
MOL005503	Cornudentanone	39.7	0.327	SHANZHUYU
MOL005530	Hydroxygenkwanin	36.5	0.272	SHANZHUYU
MOL005531	Telocinobufagin	70.0	0.793	SHANZHUYU
MOL005552	gemin D	68.8	0.561	SHANZHUYU
MOL005557	lanosta-8,24-dien-3-ol,3-acetate	44.3	0.824	SHANZHUYU
MOL005944	matrine	63.8	0.249	TUSIZI
MOL006209	cyanin	47.4	0.759	GOUQIZI
MOL006649	sophranol	55.4	0.282	TUSIZI
MOL007449	24-methylidenelophenol	44.2	0.753	GOUQIZI
MOL008173	daucosterol_qt	36.9	0.753	GOUQIZI
MOL008400	glycitein	50.5	0.238	GOUQIZI
MOL008457	Tetrahydroalstonine	32.4	0.813	SHANZHUYU
MOL009604	14b-pregnane	34.8	0.337	GOUQIZI
MOL009612	(24R)-4alpha-Methyl-24-ethylcholesta-7,25-dien-3beta-ylacetate	46.4	0.840	GOUQIZI
MOL009615	24-Methylenecycloartan-3beta,21-diol	37.3	0.798	GOUQIZI
MOL009617	24-ethylcholest-22-enol	37.1	0.751	GOUQIZI
MOL009618	24-ethylcholesta-5,22-dienol	43.8	0.756	GOUQIZI
MOL009620	24-methyl-31-norlanost-9 (11)-enol	38.0	0.751	GOUQIZI
MOL009621	24-methylenelanost-8-enol	42.4	0.768	GOUQIZI
MOL009622	Fucosterol	43.8	0.757	GOUQIZI
MOL009631	31-Norcyclolaudenol	38.7	0.814	GOUQIZI
MOL009633	31-norlanost-9 (11)-enol	38.4	0.725	GOUQIZI
MOL009634	31-norlanosterol	42.2	0.730	GOUQIZI
MOL009635	4,24-methyllophenol	37.8	0.750	GOUQIZI
MOL009639	Lophenol	38.1	0.714	GOUQIZI
MOL009640	4alpha,14alpha,24-trimethylcholesta-8,24-dienol	38.9	0.758	GOUQIZI
MOL009641	4alpha,24-dimethylcholesta-7,24-dienol	42.7	0.753	GOUQIZI
MOL009642	4alpha-methyl-24-ethylcholesta-7,24-dienol	42.3	0.783	GOUQIZI
MOL009644	6-Fluoroindole-7-Dehydrocholesterol	43.7	0.722	GOUQIZI
MOL009646	7-O-Methylluteolin-6-C-beta-glucoside_qt	40.8	0.305	GOUQIZI
MOL009650	Atropine	42.2	0.193	GOUQIZI
MOL009651	Cryptoxanthin monoepoxide	47.0	0.561	GOUQIZI
MOL009653	Cycloeucalenol	39.7	0.794	GOUQIZI
MOL009656	(E,E)-1-ethyl octadeca-3,13-dienoate	42.0	0.194	GOUQIZI
MOL009660	methyl (1R,4aS,7R,7aS)-4a,7-dihydroxy-7-methyl-1-[(2S,3R,4S,5S,6R)-3,4,5-trihydroxy-6-(hydroxymethyl)oxan-2-yl]oxy-1,5,6,7a-tetrahydrocyclopenta [d]pyran-4-carboxylate	39.4	0.466	GOUQIZI
MOL009662	Lantadene A	38.7	0.574	GOUQIZI
MOL009664	Physalin A	91.7	0.272	GOUQIZI
MOL009665	Physcion-8-O-beta-D-gentiobioside	43.9	0.624	GOUQIZI
MOL009677	lanost-8-en-3beta-ol	34.2	0.740	GOUQIZI
MOL009678	lanost-8-enol	34.2	0.742	GOUQIZI
MOL009681	Obtusifoliol	42.6	0.757	GOUQIZI
MOL010234	delta-Carotene	31.8	0.546	GOUQIZI
MOL012286	Betavulgarin	68.7	0.394	CHUANNIUXI
MOL012298	Rubrosterone	32.7	0.466	CHUANNIUXI
MOL012888	Citrostadienol	43.3	0.790	GOUQIZI

### ZGP active component-target gene interaction network

**FIGURE 2:**
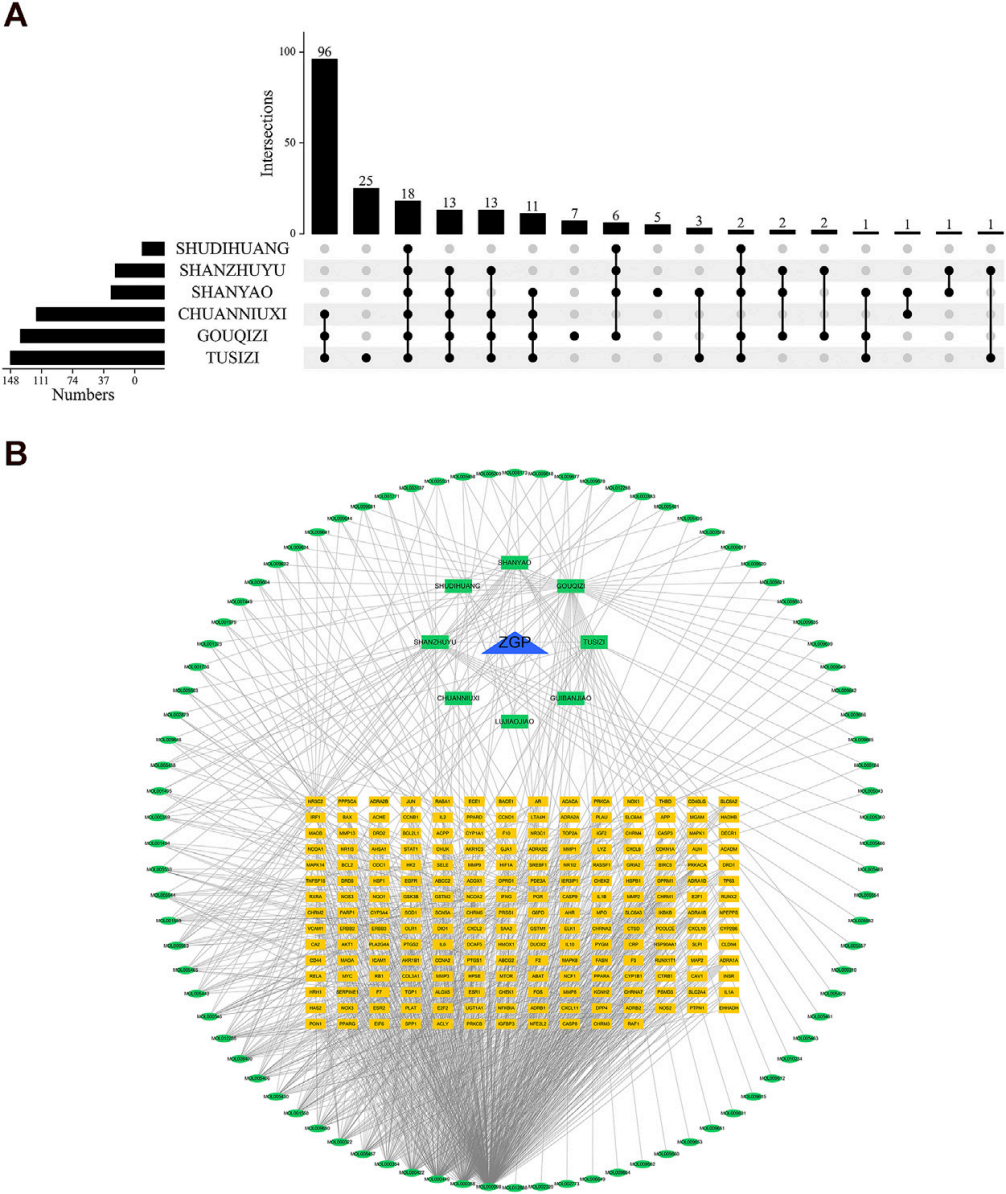
The compound-target network and analysis of ZGP. **(A)** Upset plot; **(B)** The potential targets are depicted by the yellow rectangles, the herbs by the green rectangles, the compounds by the green ellipses, and ZGP by the blue triangle.

TCMSP and PubChem gathered the 86 active components for target gene prediction. SDH (29), SY (121), SZY (112), CNX (182), GQZ (311), and TSZ (286) were among the 1041 predicted targets that were discovered. 207 relevant human gene targets were found after eliminating gene targets for other species and duplicate values. The relationship between drug composition and target was shown in Figure 2A. An herb-constituent-target network of ZGP was developed with the use of Cycloscape 3.7.1 program to make clear the relationships among the herbs. The outcomes are introduced in Figure 2B. The “CytoNCA” function was used to sort out the core components whose DC, BC, and CC were higher than the average during the topological properties analysis. Degree and intermediate centrality analyses yielded eleven core active compounds, including quercetin (MOL000098), beta sitosterol (MOL000358), stigmasterol (MOL000449), kaempferol (MOL000422), isorhamnetin (MOL000354), tetrahydroalstone (MOL008457), kadsurenone (MOL000322), sesamin (MOL001558), atropine (MOL009650), glyctein (MOL008400) and diosgenin (MOL000546).

### Potential therapeutic targets of ZGP for ischemic stroke

**FIGURE 3:**
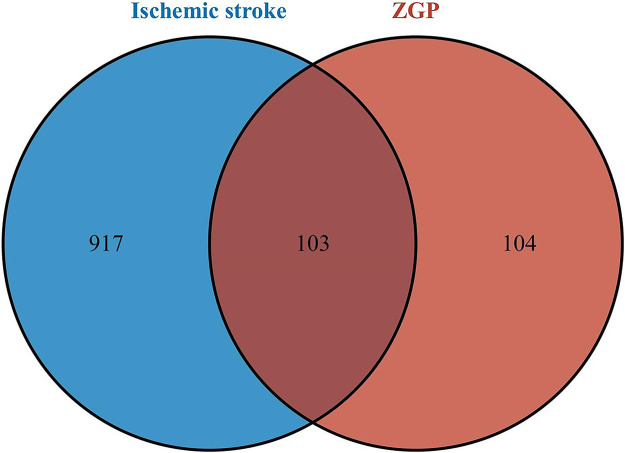
Venn diagram. The intersection of the potential ZGP targets and ischemic stroke targets.

We continued to investigate their potential therapeutic targets after clarifying the key compounds of ZGP. 1020 ischemic stroke-related targets were gathered from the TTD, OMIM, GeneCards, and DisGeNet databases after duplicates were removed. The overlapping targets of ZGP-related targets and ischemic stroke-related targets were regarded as potential therapeutic targets for ZGP anti-ischemic stroke. Screening drug component targets and ischemic stroke targets yielded 103 intersection targets, as depicted in Figure 3.

### Construction of drug component target disease network

**FIGURE 4:**
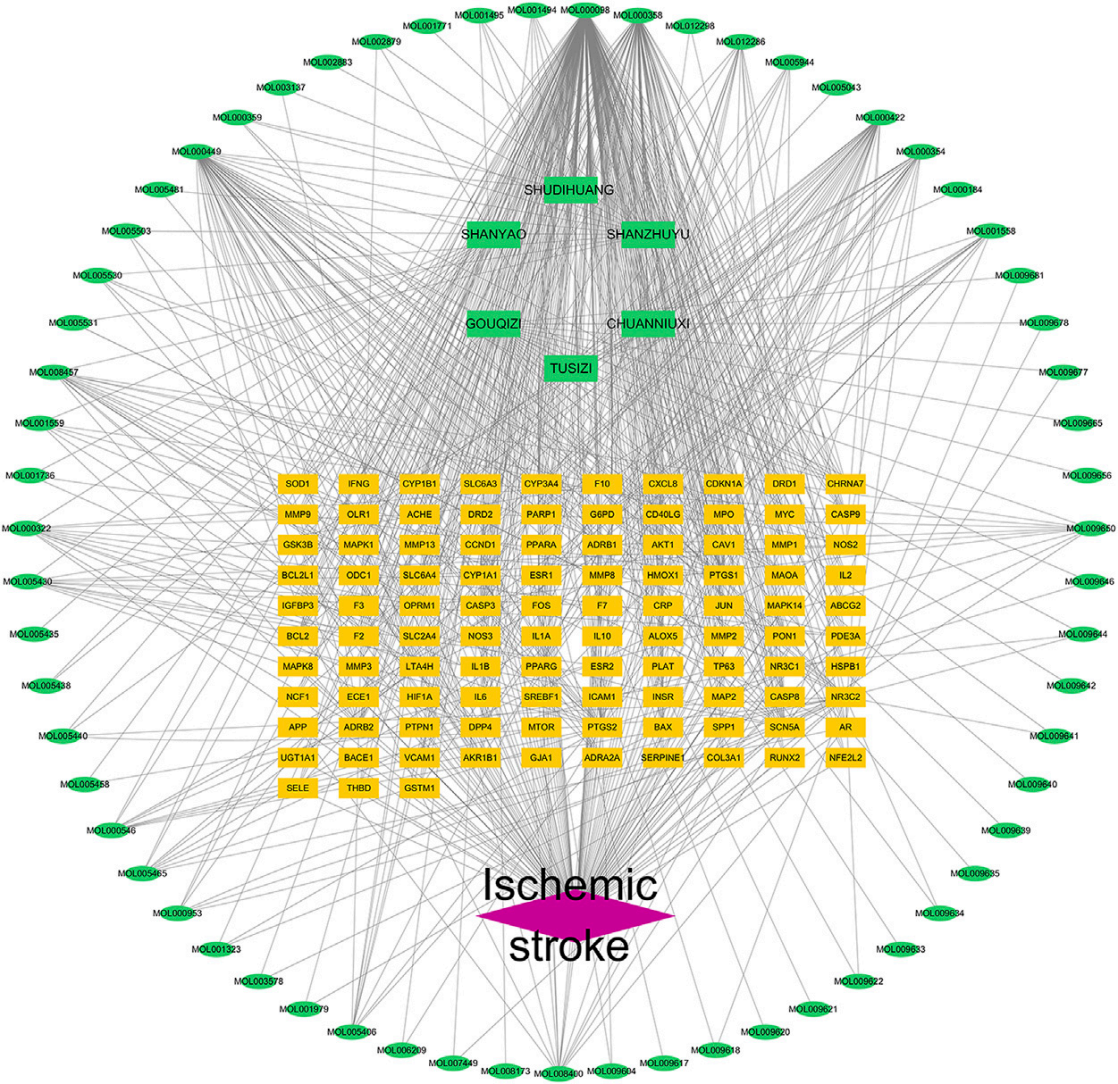
Drug component target disease network of ZGP. Network of the targets shared by ZGP and ischemic stroke. The ischemic stroke is represented by the red diamond, the potential targets are represented by the yellow rectangles, the herbs are represented by the green rectangles, and the compounds are represented by the green ellipses.

The disease-component-target network was set up in view of the strength of the 103 shared targets recognized as both potential ZGP targets and ischemic stroke targets. Figure 4 shows the 173 nodes and 720 edges in this interaction network. Quercetin, β-sitosterol, Stigmasterol, Kaempferol, and Isorhamnetin have been related with more than 15 genes among the compounds that can interact with targets related to ischemic stroke. In addition, more than ten components were linked to the genes encoding PTGS2, NR3C2, PTGS1, SCN5A, ADRB2, PPARG, DPP4, and F2. The comprehensive regulation features of multi-components and multi-targets were demonstrated by means of the disease-compound-target network.

### PPI network construction

**FIGURE 5:**
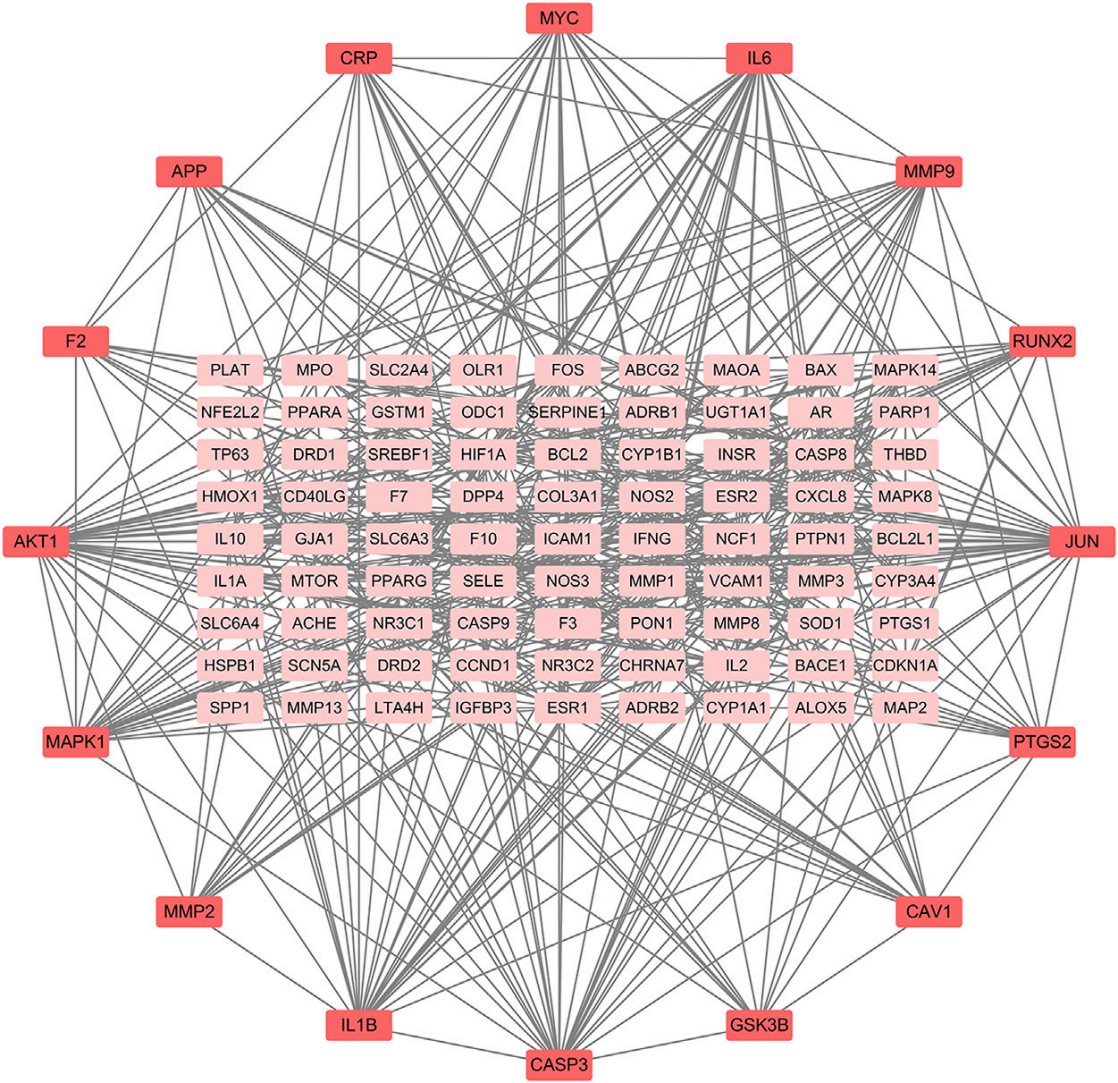
ZGP PPI networks in ischemic stroke treatment. Proteins are signified by network nodes; The targets’ abbreviations are written beside the nodes, and the straight lines represent their connections. The correlation is greater the darker the color.

The PPI relationships of 103 target genes were obtained using the STRING platform to make clear the potential mechanism by which ZGP benefits ischemic stroke. The outcomes are depicted in Figure 5. The PPI network had 475 edges and 97 nodes. The “CytoNCA” function was used to analyze the topological properties and select the key targets whose DC, BC, and CC were higher than the average. Table 2 displays a total of 16 key targets that have been obtained.

**TABLE 2:** Core targets in the PPI network.

Name	Target	DC	BC	CC
AKT1	RAC-alpha serine/threonine-protein kinase	41	1113.8	0.4948
JUN	Transcription factor AP-1	36	987.1	0.5134
IL6	Interleukin-6	32	635.5	0.4706
CASP3	Caspase-3	28	463.5	0.4660
IL1B	Interleukin-1 beta	28	501.8	0.4528
MAPK1	Mitogen-activated protein kinase 1	25	833.1	0.4848
MYC	Myc proto-oncogene protein	21	374.0	0.4305
MMP9	Matrix metalloproteinase-9	21	498.7	0.4593
PTGS2	Prostaglandin G/H synthase 2	17	562.5	0.4305
CAV1	Caveolin-1	17	446.6	0.3967
RUNX2	Runt-related transcription factor 2	16	245.2	0.4192
CRP	C-reactive protein	15	276.0	0.3887
GSK3B	Glycogen synthase kinase-3 beta	13	213.1	0.4085
MMP2	72 kDa type IV collagenase	13	223.8	0.4211
APP	Amyloid beta A4 protein	13	922.9	0.4156
F2	Prothrombin	12	830.8	0.4229

### Analysis of GO and KEGG enrichment

**FIGURE 6:**
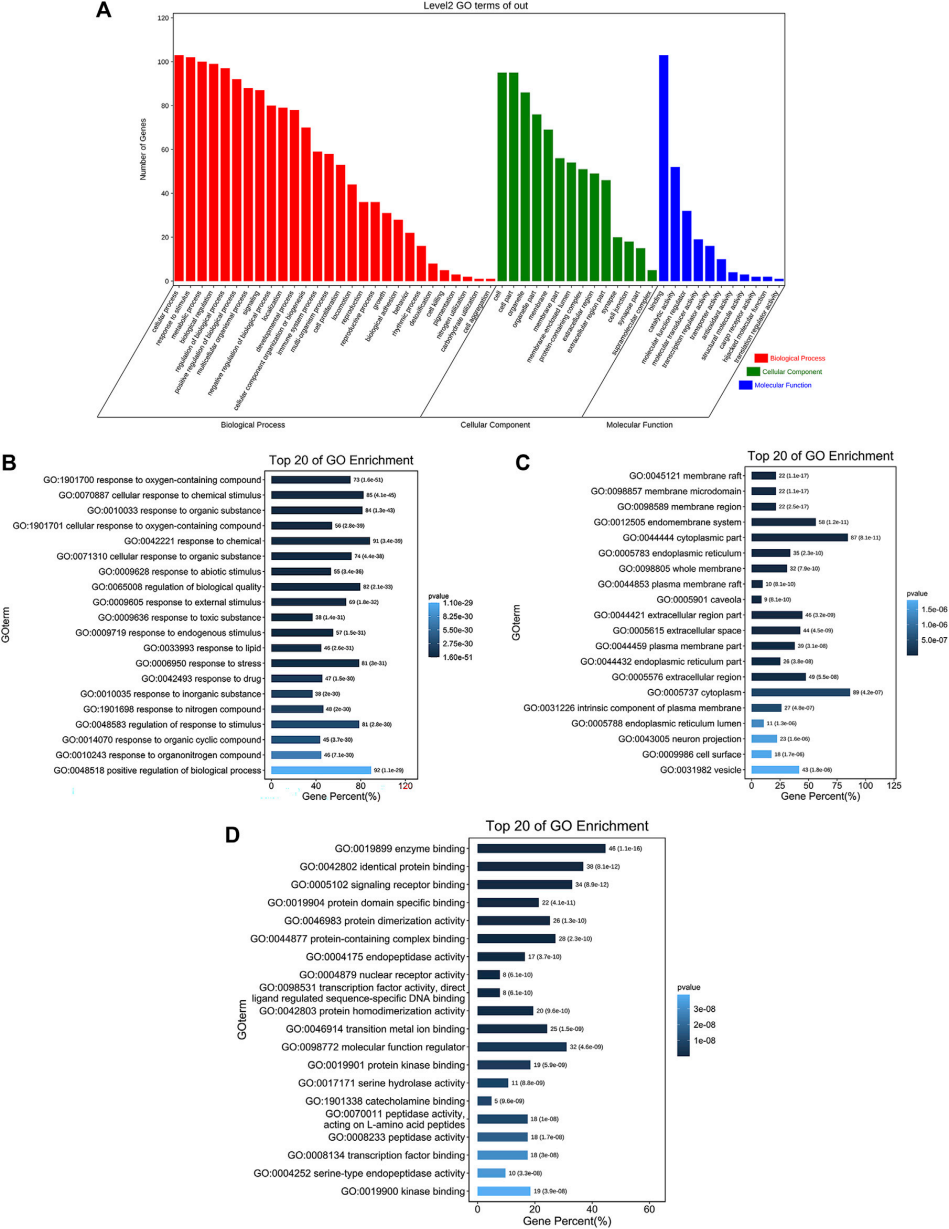
The findings of GO enrichment analysis. **(A)** On top of each bar was the number of genes in each category. The corresponding biological process (BP), cellular component (CC), and molecular function (MF) are represented by the colors red, green, and blue. **(B)** The top 20 of biological process. **(C)** The top 20 of cellular component. **(D)** The top 20 of molecular function.

**FIGURE 7:**
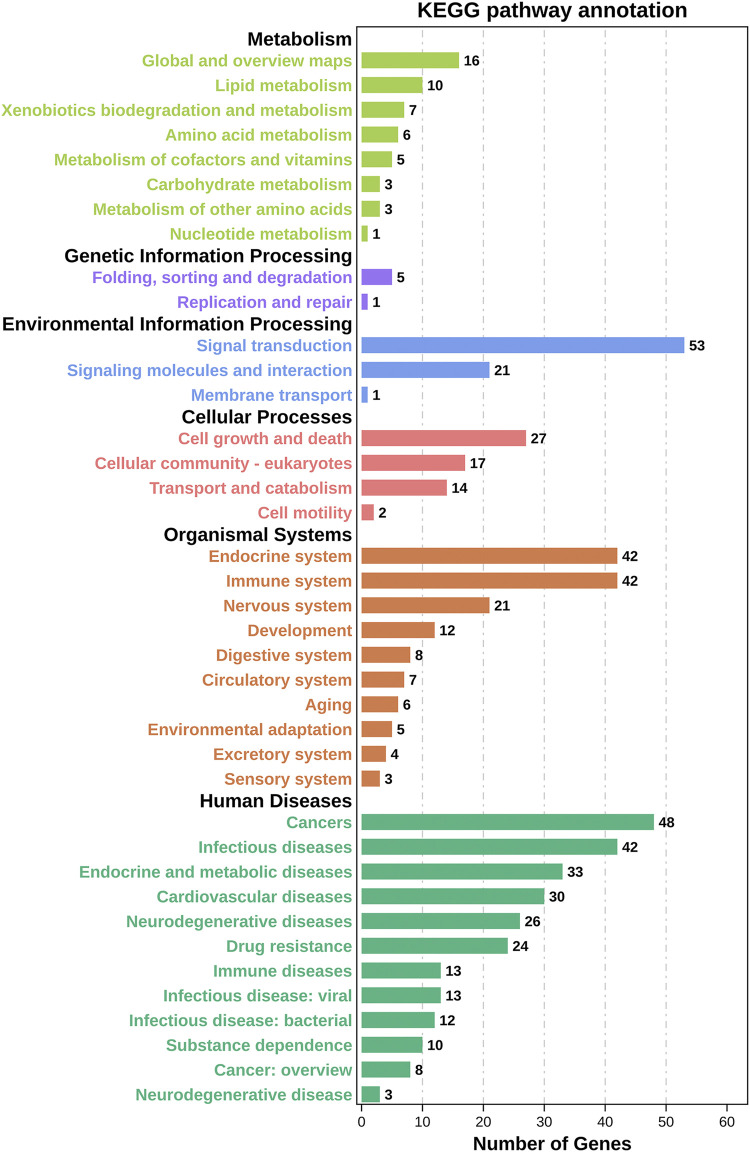
Results of KEGG enrichment analysis. On top of each bar was the number of genes in each category.

**FIGURE 8:**
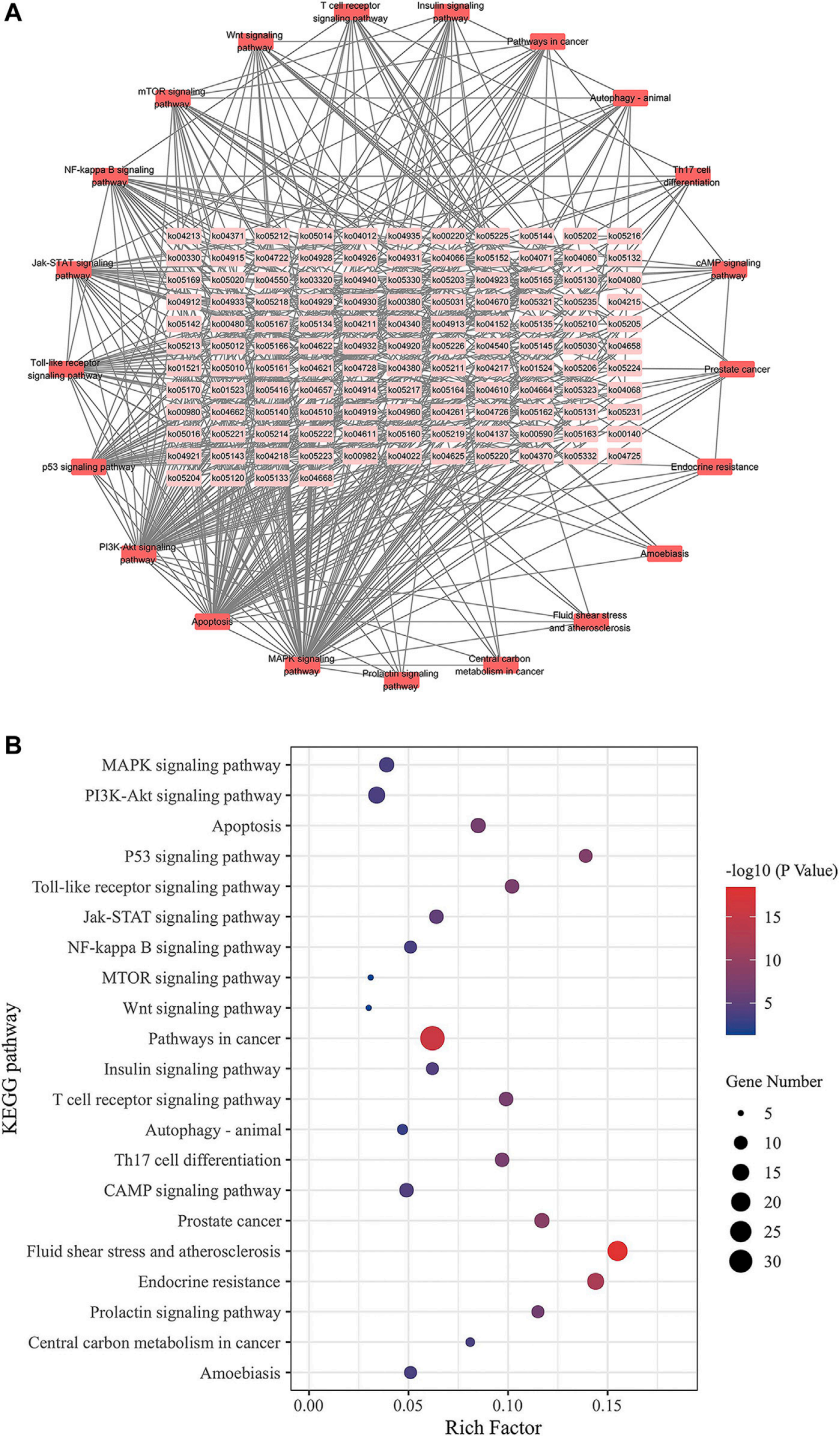
KEGG pathway interaction network. **(A)** Pathways are represented by network nodes; The annotates of the pathways are written on the nodes. Associations between the pathways are represented by straight lines. The correlation is stronger when the hue is darker. **(B)** Ischemic stroke-related key pathways.

We inserted the aforementioned targets into the OmicShare evaluation platform for GO and KEGG enrichment analyses in order to gain a deeper comprehension of the achievable pharmacological activity of ZGP in the treatment of ischemic stroke. 3569 biological processes (BPs), 228 cellular components (CCs), and 350 molecular functions (MFs) were among the 4147 GO terms we obtained (Figure 6A). Figures 6B–D depict the top 20 BP, CC, and MF categories. The targets had the highest GO enrichment in the following biological process ontologies: response to an organic substance, cellular response to a chemical stimulus, and oxygen-containing compound. Membrane raft, membrane microdomain, membrane region, endomembrane system, and cytoplasmic part were among the most highly enriched cellular component ontologies. ZGP’s synergistic effects covered binding of enzymes, identical protein, signaling receptors, and other molecular functions. 149 signaling pathways have been recognized via enrichment and screening of KEGG pathways (*p* < 0.05) (Figure 7). The KEGG pathway interaction network was established for the enrichment results of KEGG pathways (Figure 8A), and 21 key pathways related to ischemic stroke through using the “CytoNCA” function to analyze its topological properties (Figure 8B; Table 3). The main signaling pathway included MAPK, PI3K-Akt, Apoptosis, p53, Toll-like receptor, Jak-STAT, NF-kappa B, mTOR, and Wnt signaling pathway.

**TABLE 3:** 21 key pathways related to ischemic stroke in KEGG pathway interaction network.

ID	Pathway	DC	BC	CC
ko04010	MAPK signaling pathway	82	7167.5	0.6682
ko04151	PI3K-Akt signaling pathway	62	3451.7	0.6067
ko04210	Apoptosis	62	4839.3	0.6092
ko04115	p53 signaling pathway	34	1012.0	0.5124
ko04620	Toll-like receptor signaling pathway	32	1111.0	0.5124
ko04630	Jak-STAT signaling pathway	31	612.5	0.5179
ko04064	NF-kappa B signaling pathway	28	654.7	0.4849
ko04150	mTOR signaling pathway	24	386.4	0.4770
ko04310	Wnt signaling pathway	18	250.0	0.4531
ko05200	Pathways in cancer	15	296.6	0.4817
ko04910	Insulin signaling pathway	15	375.0	0.4739
ko04660	T cell receptor signaling pathway	15	323.9	0.4517
ko04140	Autophagy - animal	14	433.3	0.4421
ko04659	Th17 cell differentiation	10	219.0	0.4421
ko04024	cAMP signaling pathway	10	259.8	0.4448
ko05215	Prostate cancer	9	717.0	0.4708
ko05418	Fluid shear stress and atherosclerosis	6	236.4	0.4545
ko01522	Endocrine resistance	6	345.3	0.4574
ko04917	Prolactin signaling pathway	6	634.1	0.4367
ko05230	Central carbon metabolism in cancer	6	444.7	0.4315
ko05146	Amoebiasis	6	290.1	0.4155

### Molecular docking

**FIGURE 9:**
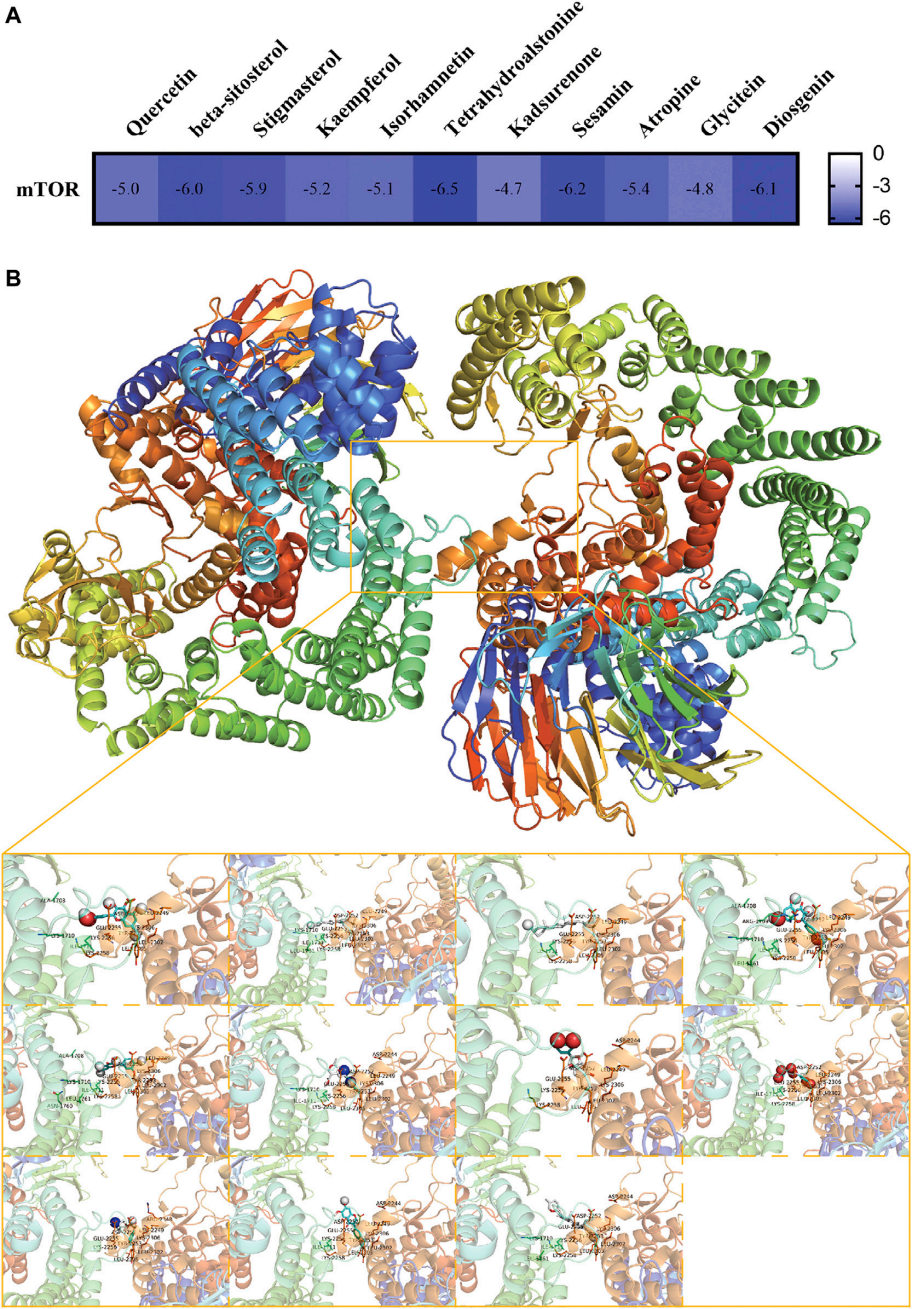
Molecular Docking Results. **(A)** The affinity of optimal docking results; **(B)** The visualization of optimal docking results (Middle: mTOR protein and its docking pocket. From left to right, top to bottom: Quercetin, beta-sitosterol, stigmasterol, kaempferol, isorhamnetin, tetrahydroalstonine, kadsurenone, sesamin, atropine, glyctein and diosgenin).

Eleven active compounds (Quercetin, beta-sitosterol, stigmasterol, kaempferol, isorhamnetin, tetrahydroalstonine, kadsurenone, sesamin, atropine, glyctein and diosgenin) were obtained by network pharmacological analysis as the core active compounds of ZGP in the treatment of ischemic stroke. Take mTOR as the potential target. Through docking simulations, the results were shown in Figure 9A. The molecular-docking results suggested that the core active compounds of ZGP had good inter binding with mTOR. Detailed information about the optimal docking modes of the active compounds with mTOR were shown in Figure 9B.

### ZGP increased the cell viability of differentiated SH-SY5Y cells after OGD/R

**FIGURE 10:**
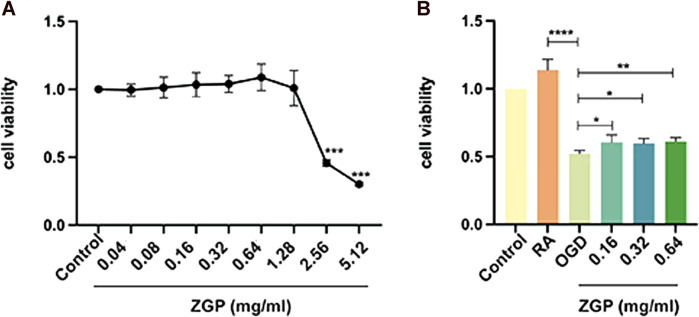
Impacts of ZGP on cell viability. The CCK-8 test was used to determine the cell viability, which was expressed as the OD value. **(A)** The cytotoxic effects of ZGP with different concentrations on differentiated SH-SY5Y cells. **(B)** ZGP increased the cell viability of differentiated SH-SY5Y cells after OGD/R. Each point denotes mean ± SD (n = 6). *p < 0.05, compared with OGD cohort; **p < 0.01, compared with OGD cohort; ****p < 0.0001, compared with RA cohort.

The relative cell viability was evaluated using the CCK-8 test. SH-SY5Y cells were induced to differentiate into mature neurons by ATRA. First, we evaluated ZGP’s cytotoxic effects on differentiated SH-SY5Y cells. ZGP was applied to differentiated SH-SY5Y cells at varying concentrations (0.14–5.12 mg/mL) over 24 h; the highest nontoxic concentration was 1.28 mg/mL (Figure 10A). The concentration range at 0.16 mg/mL, 0.32 mg/mL, and 0.64 mg/mL, which demonstrated the best activity, was chosen for the future testing. Differentiated SH-SY5Y cells were treated by OGD for 4 h. The cells were supplemented with ZGP at a concentration of 0.16 mg/mL, 0.32 mg/mL, and 0.64 mg/mL for 24 h. The ZGP cohorts had higher cell viability than the OGD cohort, and the difference between the ZGP cohorts (0.16 mg/mL, 0.32 mg/mL and 0.64 mg/mL) and the OGD cohort were statistically significant (*p* < 0.05, *p* < 0.05 and *p* < 0.01, respectively) (Figure 10B). These results demonstrated that differentiated SH-SY5Y cells were protected from OGD/R damage by ZGP.

### ZGP promoted neurite outgrowth of differentiated SH-SY5Y cells after OGD/R

**FIGURE 11:**
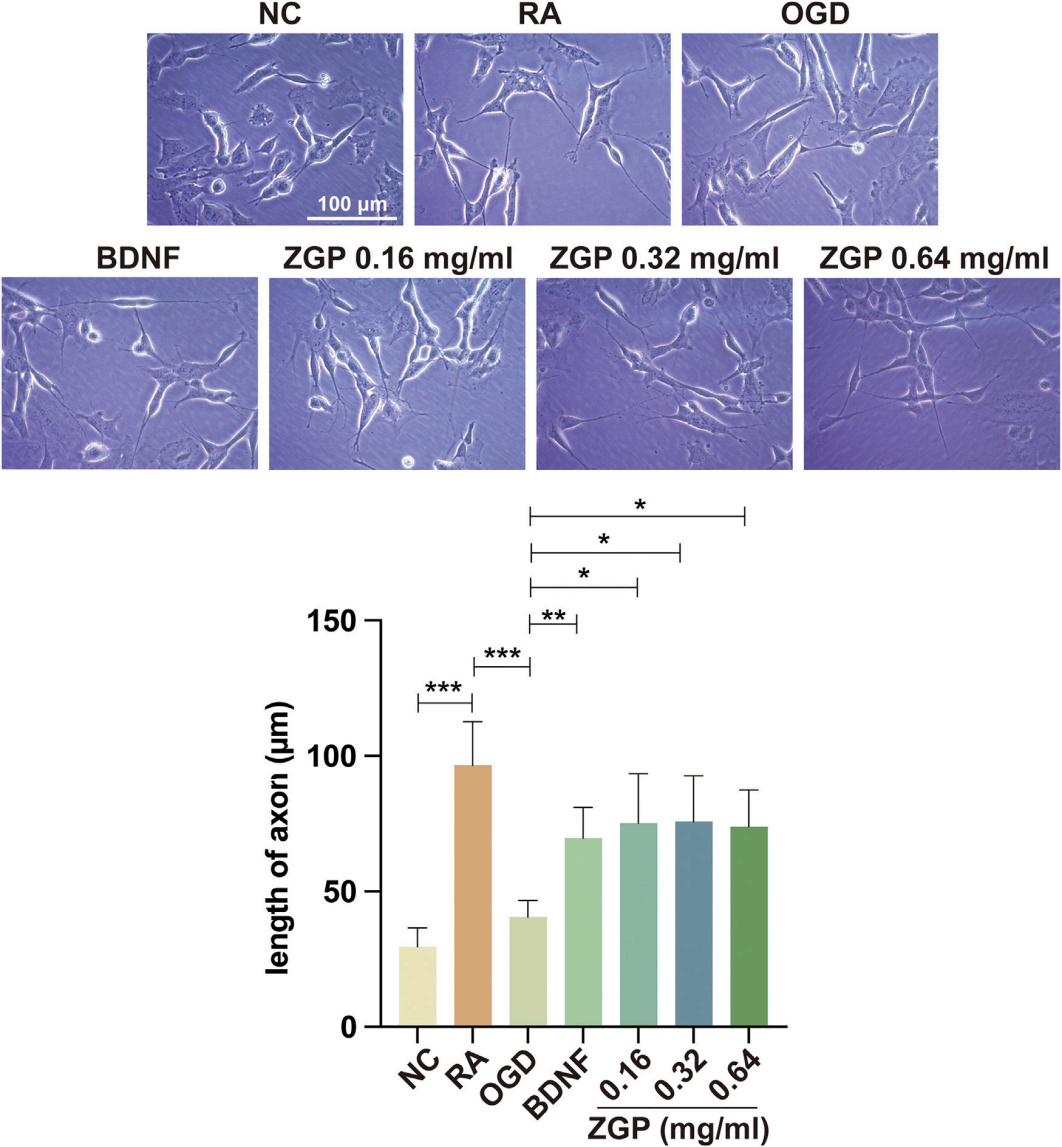
Effects of ZGP on neurite outgrowth. Illustrative microphotographs of morphological characteristics **(A)** and quantitative measurement outcomes of neurites length **(B)** of the normal control, differentiated SH-SY5Y cells between the OGD and ZGP cohorts 3 days after treatment; When compared to the OGD cohort, the cohorts with various concentrations of ZGP (0.16 mg/mL, 0.32 mg/mL, 0.64 mg/mL) had significantly longer neurites; each point represents mean ± SD (n = 3). ****p < 0.0001, compared with OGD cohort; scale bars: 100 μm.

The neurite alteration of neural cells was observed 3 days after treatment to determine the effect of ZGP on the neurite outgrowth of differentiated SH-SY5Y cells. Positive control was BDNF with 50 ng/mL, which is known to induce neurite outgrowth and promote synaptic plasticity. As depicted in Figure 11A, the cell morphology in the normal control group was irregular quadrilateral and elliptical, and the neurites were very short. In the ATRA induced group, the neurites of cells were much longer than those of immature cells, and the length of axons of some differentiated cells could reach 100–120 μm. The number of cells in the OGD cohort decreased, the cell body became flat, and the axons shortened compared with the ATRA induced group (Figure 11A). The OGD cohort’s neurites were drastically shorter than those of the ATRA-induced group (*p* < 0.0001). As was to be expected, BDNF significantly improved the morphology of damaged cells and prolonged neurites growth. Meanwhile, the length of neurites was significantly longer after supplementation with various concentrations of ZGP (0.16, 0.32, 0.64 mg/mL) compared to that in the OGD cohort (*p* < 0.0001, *p* < 0.0001, *p* < 0.0001) (Figure 11B). When compared to the OGD cohort, the impact of ZGP on neurite outgrowth was comparable to that of the positive control BDNF. The result suggested that ZGP reversed the damage to differentiated SH-SY5Y cells induced by OGD/R and promoted neurite or axon outgrowth.

### ZGP regulated GAP43 and PTEN/mTOR/p-S6 signaling pathway proteins expression

**FIGURE 12:**
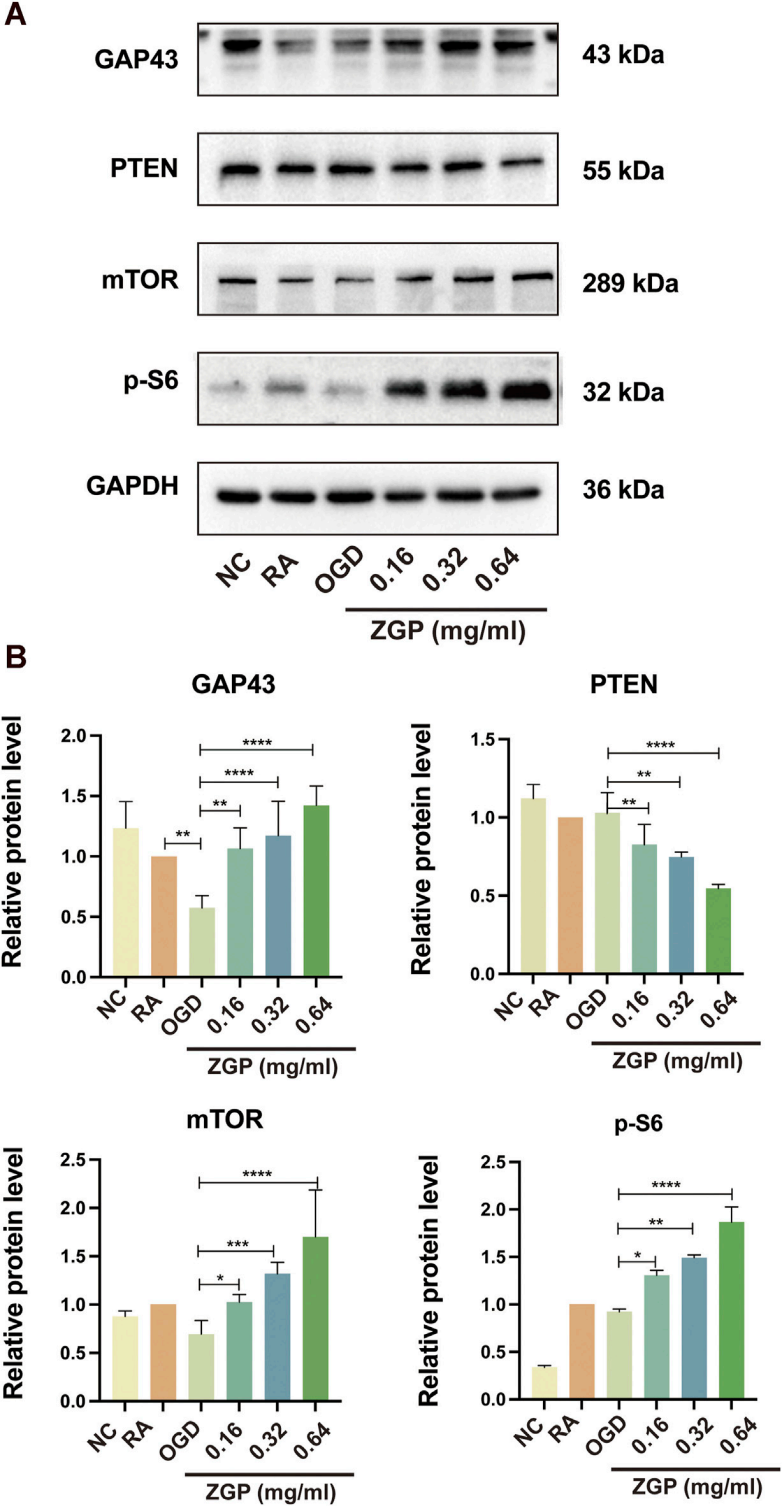
Effect of ZGP on GAP43, PTEN, mTOR, and p-S6 proteins expression. The differentiated SH-SY5Y cells were planted in 100-mm dishes and supplemented with various concentrations of ZGP (0.16 mg/mL, 0.32 mg/mL, 0.64 mg/mL) for 72 h; **(A)** the levels of GAP43, PTEN, mTOR, and p-S6 proteins expression were identified by Western blotting; **(B)** the expression of the GAP43, PTEN, mTOR, and p-S6 proteins in cells was quantified by normalizing to GAPDH; The data are shown as the means ± SD (n = 3). *p < 0.05, **p < 0.01, ***p < 0.001, ****p < 0.0001, compared with OGD cohort.

Based on the results of GO and KEGG enrichment analysis, we selected the mTOR signal pathway protein and its negative regulatory factor PTEN protein for Western blot detection to verify the mechanism of ZGP promoting neurite growth. The expression of axon growth marker protein GAP43 in induced SH-SY5Y cells decreased after OGD/R (*p* < 0.0001), but gradually increased with the increase of ZGP concentration (Figure 12A). We observed statistically significant differences in 0.16 mg/mL (*p* < 0.01), 0.32 mg/mL (*p* < 0.0001) and 0.64 mg/mL cohort (*p* < 0.0001) compared to that in OGD cohort (Figure 12B). The expression of mTOR and p-S6 also had similar changes with GAP43 in various concentrations of ZGP (0.16 mg/mL, 0.32 mg/mL, 0.64 mg/mL) cohorts, which increased dramatically compared with the OGD cohort (*p* < 0.05–0.0001). Compared to the OGD cohort, PTEN protein expression was significantly downregulated in the ZGP cohorts at various concentrations (0.16 mg/mL, 0.32 mg/mL, 0.64 mg/mL) (*p* < 0.01, *p* < 0.01, *p* < 0.0001). These findings suggest that ZGP increased the expression of the axon-related protein GAP43 and promoted the growth of neuronal axons. ZGP’s mechanism may involve increasing mTOR and p-S6 protein expression while decreasing that of the negative regulator PTEN protein.

## Discussion

The incidence of ischemic strokes in China has increased by 86.0% since 1990, with 3.94 million new cases annually (Ma et al., 2021). Researchers have demonstrated that ZGP can significantly improve the neurological function of patients or mice who have suffered an ischemic stroke in clinical trials and basic experiments (Li et al., 2019; Liu et al., 2022). The total effective rate of ZGP for stroke patients was 92.0% (Chen et al., 2015). Using network pharmacology methods and a series of experiments, we determined the efficacy and molecular mechanisms of ZGP on ischemic stroke.

There were 86 active ingredients and 207 compound-related targets verified in ZGP in this study, and 1020 targets related to ischemic stroke were confirmed in the database. A total of 107 of these targets were related to ischemic strokes and ZGP. Additionally, 11 core active compounds (Quercetin, beta sitosterol, stigmasterol, kaempferol, isorhamnetin, tetrahydroalstone, kadsurenone, sesamin, atropine, glyctein and diosgenin) were obtained by network pharmacological analysis. Quercetin exerts neuroprotection for acute ischemic stroke by improving neurological function score (NFS), reducing infarct volume, and protecting blood brain barrier (BBB) integrity (Guo et al., 2022). There are several mechanisms by which quercetin protects the brain from damage, namely antioxidant, anti-inflammatory, anti-apoptosis, and the ability to resist calcium overload (Wang et al., 2020). Moreover, Quercetin might upregulate expression of GAP43, promote neurite outgrowth and regeneration of DRGs neurons and PC12 cells (Chen et al., 2015; Katebi et al., 2019), enhance the proliferation and migration of Schwann cells, improve locomotor function recovery, axonal regeneration and energy metabolism after spinal cord injury (SCI) (Wang et al., 2020). As naturally occurring sterol compounds, beta-sitosterol and stigmasterol play an important in cholesterol homeostasis, antioxidation activity, anti-inflammation activity, and nervous system development (Gao et al., 2021). There is evidence that stigmasterol protects against ischemic and reperfusion injury through its actions on AMPK/mTOR and JNK signaling pathways, which reduce oxidative stress and inactivate autophagy (Sun et al., 2019). The genes involved in the formation of neurites and synaptic transmission could be upregulated by stigmastero (Haque et al., 2018). It has been demonstrated that Stigmasterol modulated both pre-and post-synaptic events after ischemic and reperfusion, especially by attenuating GluN2B-mediated excitotoxicity and oxidative stress, and inducing mitophagy (Haque et al., 2021). β-sitosterol and stigmastero were also found to have neurite outgrowth-promoting activity in PC12 cells, which was induced by enhancing NGF and neurofilament expression (Koga et al., 2020). In addition to its anti-inflammatory and antioxidant properties, a flavonoid known as kaempferol has antibacterial and antiviral properties as well (Silva Dos Santos et al., 2021). Kaempferol has been found to exhibit neuroprotective properties during cerebral ischemia. It can prevent cell death, oxidative stress, mitochondrial malfunction, and apoptosis that are caused by oxygen-glucose deprivation (OGD) (Wang et al., 2020). Kaempferol improved neurological impairments in cerebral ischemia reperfusion rats by reducing neuroinflammation and blood brain barrier dysfunction; the NF-B pathway was involved in this process (Li et al., 2019). Through increasing the levels of brain-derived neurotrophic factor (BDNF), kaempferol is also crucial for memory, neuronal plasticity, and the development of new neural networks (Yan et al., 2019). Isorhamnolicin has the effect of promoting neurite outgrowth and may drive PC12 cells to differentiate into neural cells (Xu et al., 2012). It was discovered that administering isorhamnetin to experimental ischemic mice decreased infarct volume and caspase-3 activity, reduced cerebral edema, protected blood-brain barrier, and accelerated the recovery of neurological function (Zhao et al., 2016). Sesamin is a lignan that has the ability to delay aging, resist oxidation and apoptosis, regulate oxidative stress, and diminish inflammatory response (Rao et al., 2018). Due to its extensive spectrum of pharmacological effects and therapeutic qualities, diosgenin, a well-known steroidal sapogenin, has been utilized for the treatment of neurological illnesses such cerebrovascular disease, Parkinson’s disease, Alzheimer’s disease, and brain damage (Cai et al., 2020). Enhanced expression of nerve growth factor (NGF) was related to greater neurite outgrowth, repaired damaged axons, restored ultrastructural alterations, and neuronal regeneration in a diabetic mice model (Kang et al., 2011). According to these studies, ZGP’s primary ingredients are useful for treating ischemic stroke since they not only protect the brain from damage but also encourage nerve regeneration and repair. The active ingredient-target network diagram demonstrates ZGP’s multiple constituents and multiple targets. PPI system analysis identified a total of 16 critical targets genes, with AKT1, JUN, IL6, and CASP3 placing at the top of the list. Serine/threonine protein kinase known as AKT1 is involved in a variety of physiological and pathological processes, including cell differentiation, apoptosis, inflammation, and metabolism following ischemia (Zhao et al., 2016; Samakova et al., 2019). Activated AKT1 triggers a series of signal cascade reactions, which can reduce the death of brain cells, promote the growth of neural cells and vascular endothelial cells, enhance the regeneration and repair of nerve tissue and vessels, and improve neural function after cerebral ischemia (Huang et al., 2021; Wang et al., 2021). Researches show that the expression of Jun gene is related to nerve regeneration, participate in the processes of neurovascular remodeling and recovery after cerebral ischemia (Murata et al., 2012). Whether in peripheral nerve tissue or central nerve tissue, the expression of Jun gene is induced after nerve injured. The higher the expression level and the longer the expression duration of Jun gene, the stronger the ability of nerve regeneration (Jessen and Mirsky, 2016). L6, an vital inflammatory cytokine, has many biological functions, which include regulating immune responses and inflammation (Lasek-Bal et al., 2019). After cerebral ischemia, the damaged brain tissue produces immune response and activates inflammatory cells. IL-6 is secreted to participate in secondary brain injury, increases infarct size and worsens clinical outcomes (Zhang et al., 2019). A key member of the Caspase family, Caspase-3 is the “molecular switch” that controls apoptosis in cells (Asadi et al., 2022). Caspase-3 protein expression significantly increased in the ischemic area of the brain, according to studies. The recovery of neural function and the survival of neural and vascular cells can both be enchanced by inhibiting Caspase-3 protein expression (Wesley et al., 2021).

To gain a deeper comprehension of the target genes’ interaction and action pathways, GO and KEGG pathway analyses were utilized. The following biological processes (BPs) were found to be strongly related with target genes through GO analysis: response to an organic substance, cellular response to a chemical stimulus, and oxygen-containing compound. Membrane raft, membrane microdomain, membrane region, endomembrane system, and cytoplasmic part were the most highly enriched CCs ontologies. Enzyme binding, identical protein binding, signaling receptor binding, and various other molecular functions were included in the enriched MF ontologies. The MAPK, PI3K-Akt, apoptosis, p53, Toll-like receptor, Jak-STAT, NF-kappa B, mTOR, and Wnt signaling pathways were the primary focus of the KEGG pathway analysis. The results shown that ZGP may act on multiple signal pathways.

The mitogen-activated protein kinase (MAPK) and PI3K/Akt pathways play a significant role in the activation of a number of critical signal transduction pathways in response to stress (Zheng et al., 2020; Liu et al., 2021). They not only participate in the regulation of apoptosis and inflammatory response after cerebral ischemia, but also mediates the proliferation, differentiation, and growth of neurons (Kong et al., 2016; Jayaraj et al., 2019). PI3K/Akt signal pathway is the most effective anti apoptotic pathway after the activation by granulocyte and macrophage stimulator, p-Akt can inhibit the expression of downstream apoptotic factors NF- κ B, regulates cell metabolism, proliferation, apoptosis and migration (Zhu et al., 2018). The JAK/STAT signaling pathway plays a major role in the process of apoptosis following ischemia reperfusion injury, which is abnormally activated to accelerate neuronal apoptosis and aggravate brain injury (Wu et al., 2018).

mTOR, p53, and Wnt signaling pathways are all associated with nerve regeneration and repair after ischemic brain injury. mTOR is a key signal pathway regulating the intrinsic growth ability of neurite outgrowth and axonal regeneration (Park et al., 2008; Bei et al., 2016; Liu et al., 2017; Zhang et al., 2022). The phosphorylation expression level of its downstream protein p-S6 can indirectly characterize the activity of mTOR ((Ma et al., 2021; Li et al., 2022). The ability to regenerate after ischemic brain injury is closely related to the enhancement of mTOR/p-S6 activity, which increases the expression of growth associated protein-43 (GAP-43) (Li et al., 2022). According to another research, the PI3K/Akt signaling pathway is also essential for axonal regeneration, which is accomplished via activating mTOR. (Wang et al., 2019). Meanwhile, phosphatase and tensin homology (PTEN) is an important molecular target to inhibit the internal growth ability of neurons (Liu et al., 2010). It steadily increases in expression as the nervous system develops and matures, suppresses the mTOR pathway of cellular internal growth, and dramatically lowers the capacity of mature axons to sprout and regenerate (Hausott et al., 2022). Inhibiting PTEN expression to reactivate mTOR pathway can promote significant sprouting, regeneration and functional recovery of optic nerve axons and CST axons after cerebral ischemia (He and Jin, 2016). The role of tumor suppressor p53 in regulating neurite outgrowth and axonal regeneration has been confirmed over the past decade (Di Giovanni and Rathore, 2012). As a highly conserved signal pathway in multicellular eukaryotes, Wnt protein family is crucial to neuronal differentiation, neuronal survival, axonal regeneration after ischemic stroke and other aspects at the early stage of nervous system development (Garcia et al., 2018).

From the above results, we can see that ZGP not only has anti-apoptosis and neuroprotective effects on ischemic stroke, but also can promote neurite outgrowth and axonal regeneration after cerebral ischemia. Our *in vitro* experiments in this study demonstrated that ZGP protected ischemic neurons by increasing the cell viability of differentiated SH-SY5Y cells following OGD/R. Several other investigations have revealed that the mechanism behind ZGP’s protective impact on ischemic stroke is related to inhibit oxidative stress and inflammatory reaction and regulate PI3K/Akt pathway (Liu et al., 2017; Liu et al., 2021; Liu et al., 2022). However, the effect and mechanism of ZGP on neurite growth and regeneration have not been experimentally verified. In current research, we targeted on the effect of ZGP on neurite growth and regeneration in hypoxic-ischemic neurons, and verified its mechanism. As we expected, ZGP reversed the damage to differentiated SH-SY5Y cells induced by OGD/R and promoted neurite and axon outgrowth obviously. Our previous research results and those of other scholars confirmed that PTEN/mTOR are the key signal pathway to control neurite and axon remodeling after nerve injury (Park et al., 2008; Liu et al., 2017). We verified that ZGP promotes neurite growth and regeneration through the PTEN/mTOR signaling pathway. The fact that ZGP increased GAP43, mTOR, and p-S6 expression and decreased PTEN expression indicates that the PTEN/mTOR signal pathway was connected to ZGP’s role in promoting neurite regeneration and outgrowth.

In conclusion, this research is the first time to use multiple network models to study the mechanism of ZGP on ischemic stroke, and provide new insights for the role of ZGP in ischemic stroke treatment. Network analysis of ZGP identified 86 active ingredients and 107 compound-related targets correlated with ischemic stroke. Additionally, 11 core active compounds were obtained, such as Quercetin, beta sitosterol, and stigmasterol. Most of the compounds have been proved to have pharmacological activities. Pathway enrichment demonstrated that ZGP may exert neuroprotective effect through MAPK, PI3K-Akt signaling pathway, and exert promoting neurite outgrowth and axonal regeneration effect via mTOR, p53 and Wnt signaling pathway. *In vitro* experiment, ZGP treatment greatly boosted the survivability of ischemic neurons and enhanced their capacity for neurite outgrowth. Western blot assays shown that the pro-neurite outgrowth effect of ZGP on ischemic stroke may be relate to PTEN/mTOR signal pathway. All of the findings offered fresh explanations for the molecular basis of ZGP and served as a guide for its use in clinical settings. However, due to the limited database of traditional Chinese medicine and many network prediction results, the experimental verification is not comprehensive. Future research will still need to evaluate the therapeutic effects of ZGP *in vivo* and its potential mechanisms for ischemic stroke treatment.

## Data Availability

The datasets presented in this study can be found in online repositories. The names of the repository/repositories and accession number(s) can be found in the article/supplementary material.
